# Supplementary material for: The ubiquitin ligase Ariadne-1 regulates neurotransmitter release via ubiquitination of NSF
Source: J Biol Chem. 2021 Feb 11;296:100408. doi: 10.1016/j.jbc.2021.100408 (PMC7960542; doi:10.1016/j.jbc.2021.100408)
Supplement: Table S1 [file mmc2.pdf]

The ubiquitin ligase Ariadne-1 regulates neurotransmitter release via ubiquitination of NSF

Juanma Ramírez, Miguel Morales, Nerea Osinalde, Imanol Martínez-Padrón, Ugo Mayor and Alberto Ferrús

Material included:

Page S-1 .... Figure S1: figure and legend

Page S-2 .... Table S1: legend

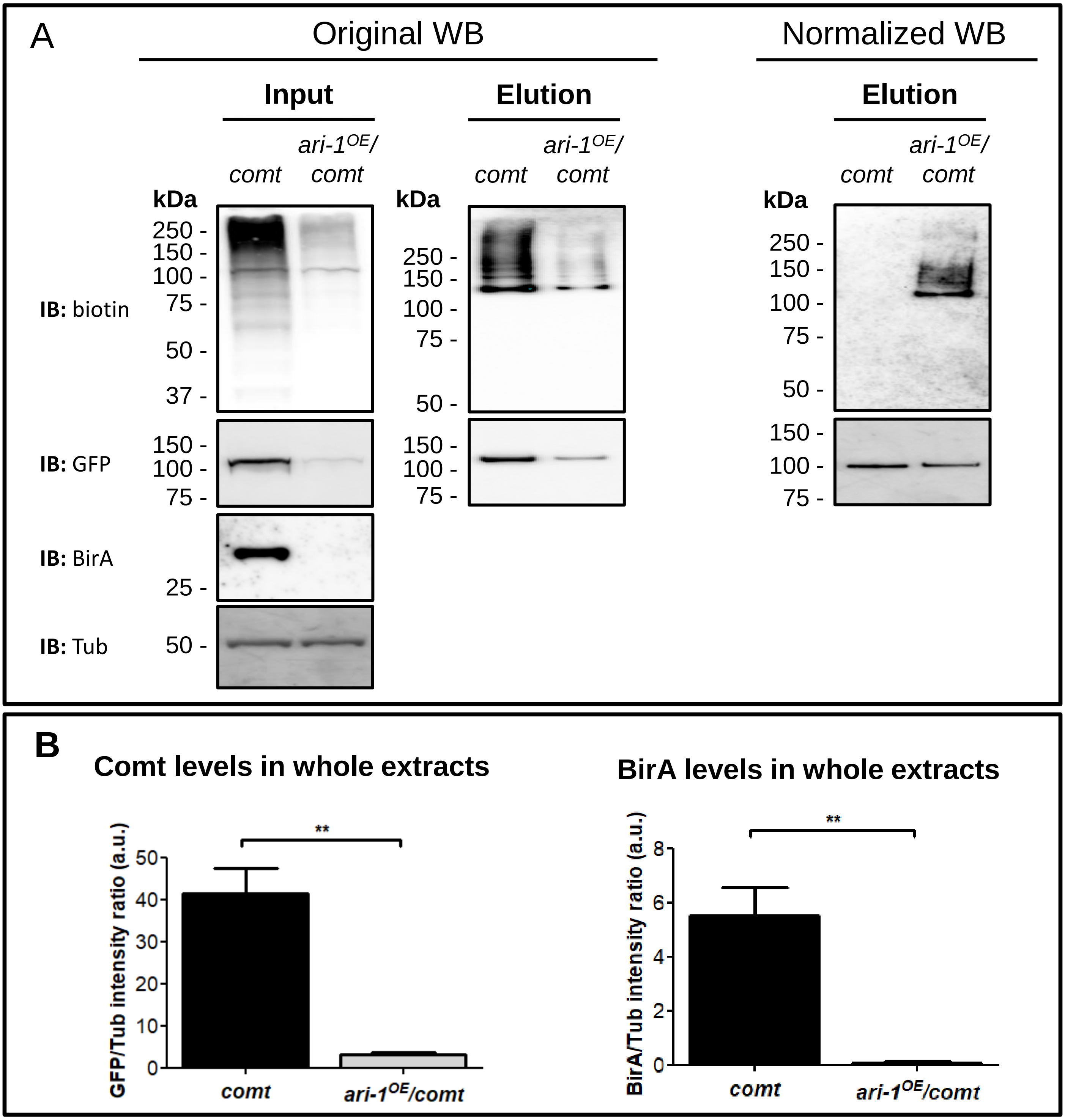

Figure S1. Ari-1 dependent ubiquitination of Comt-GFP. A) A representative immunoblot of Comt ubiquitination by Ari-1 in fly photoreceptor neurons. The ubiquitinated fraction was detected with anti-biotin antibody, while anti-GFP was used for the non-modified fraction. Anti-Tubulin was used as loading control in whole cell lysates (input). Despite Tubulin levels were similar between both genotypes, total GFP levels, as well as ubiquitin levels, were higher in *comt* flies than in *ari-1^{OE}*/*comt* flies (Original WB). This could be due to a GAL4-dose effect, as same dose of Gal4 protein is driving the expression of two UAS constructs in *comt* flies, but three UAS are present in *ari-1^{OE}/comt* flies. The fact that the levels of the (^{bio}Ub)$_6$-BirA construct, detected with anti-BirA antibody, were also lower in *ari-1^{OE}*/*comt* flies supports this idea. Comparable levels of non-modified GFP-Comt were, therefore, loaded in order to compare the ubiquitinated fraction of isolated Comt-GFP (Normalized WB). **B)** Quantification of GFP-tagged Comt and BirA levels in whole cell extract from three independent GFP pulldowns is shown. Semi-quantification of Comt-GFP and BirA was performed with Image Lab software (Bio-Rad), and their levels normalized with tubulin. Statistical significance was determined by two-tailed Student's t-test. Asterisks (**) indicates p-values equal to 0.0032 and 0.0064 for GFP and BirA, respectively.

Table S1. Mass spectrometry analysis. Proteins found more or less ubiquitinated in *ari-1*^{OE} flies, compared to control are shown in green and red, respectively (Protein Groups sheet). All GlyGly peptides identified by Mass Spectrometry (MS) are shown in the second datasheet (GlyGly peptides). Sites already reported in previous MS analysis are also indicated. Gene symbol, CG number and protein description is provided according to Flybase.
